# Implications of the evacuation of hospitalized patients in a nuclear emergency

**DOI:** 10.3389/fpubh.2023.1217118

**Published:** 2023-07-24

**Authors:** Hiroshi Yasuda

**Affiliations:** Research Institute for Radiation Biology and Medicine, Hiroshima University, Minami-ku, Hiroshima, Japan

**Keywords:** evacuation, hospitalized patients, radiation exposure, health risk, nuclear emergency, radiological accident

## Abstract

In the accident at the Fukushima Daiichi nuclear power station following the Great East Japan Earthquake and tsunami, more than 50 hospital patients died during or soon after evacuation, primarily owing to the interruption of necessary medical care. To prevent the occurrence of such losses in the future, the focus of evacuation decisions should be on the health status of individual patients and not on currently evaluated non-human aspects such as the geophysical conditions and the status of the accident facility. This brief research report provides a conceptual basis considering the principle of justification for making more appropriate decisions on the evacuation of hospitalized patients by balancing radiological risks and evacuation-induced health risks. This research report is expected to foster discussions among stakeholders on how to protect vulnerable people more appropriately in nuclear emergencies.

## 1. Introduction

The accident that occurred in March 2011 at the Fukushima Daiichi nuclear power station of Tokyo Electric Power Company Holdings, Inc (hereinafter “the Fukushima Daiichi accident”) compelled more than 150,000 residents in Fukushima Prefecture to move from their hometowns/villages in the zone within 20 km from the accident facility and the northwest area extending beyond 20 km where the radioactive deposition was significantly higher than other areas (1). During or soon after the evacuation process, more than 50 hospitalized patients died of health deterioration such as renal failure, hypothermia, and so on (2, 3). Evacuation of these inpatients and older adults in nursing homes was hastened using busses shortly after the accident and consequently, their lives were lost without excessive radiation exposure because of the interruption of necessary medical care owing to their evacuation. Furthermore, it was reported that 48% of hospitalized patients who were evacuated from selected hospitals in the affected area from 15 to 26 March 2011 died by the end of 2011 (4); in this case, parenteral administration (Hazard Ratio: 6.07) and male gender (HR: 8.35) revealed significant impacts on their mortality. In addition, nearly 1,500 evacuees aged over 65 years died approximately 1 year after the accident due to the physical and mental stress caused by changes in living conditions, including the deterioration of medical circumstances (5).

These facts indicate that evacuation can bring about significant health deterioration for some vulnerable people and, therefore, special consideration is required to sustain their medical care in the process of implementing protective measures. As it appears that this perception has not been shared adequately among all stakeholders involved in decisions regarding evacuation in a nuclear emergency, in this brief report, the author presents an understandable conceptual basis for the evacuation of hospitalized patients based on the principle of justification.

## 2. Current criteria for evacuation in a nuclear emergency

In Japan, the national guidelines for nuclear emergencies (the Nuclear Emergency Response Guidelines: NERG) were entirely revised after the Fukushima Daiichi accident and enacted in 2014, followed by subsequent amendments (6) based on international standards presented by the International Atomic Energy Agency (IAEA) (7, 8). Prefectures in Japan that have or are adjacent to nuclear facilities have updated their evacuation zone settings following this guideline (9).

**Figure 1:**
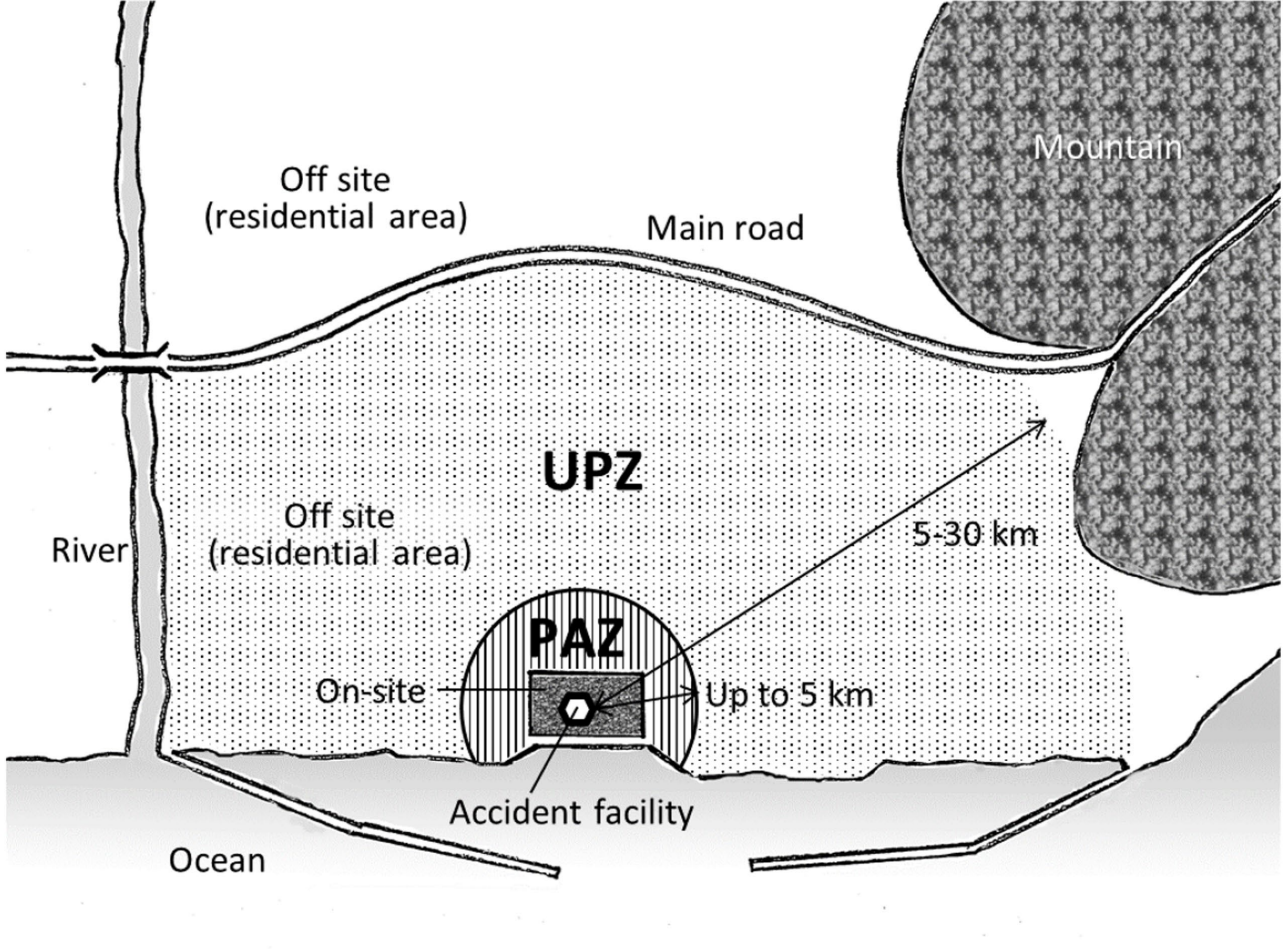
Illustration depicting the concept of the nuclear emergency planning zones formed by the Japanese government (6); here, PAZ refers to the precautionary action zone and UPZ refers to the urgent protective action planning zone (originally drawn by the author).

In this guideline (NERG), the emergency planning zone (EPZ), which was previously set within 8 to 10 km, has been reevaluated, and two zones (PAZ and UPZ) have been introduced. PAZ refers to the precautionary action zone within a distance of 5 km from the accident site where immediate protective measures, such as evacuation, should be implemented, and UPZ refers to the urgent protective action planning zone from 5 to 30 km from the accident site where some preventative protective measures, such as sheltering, would be implemented based on the state of the accident facility before radioactive materials are released. Furthermore, the NERG refers to the necessity of the measures in the area beyond 30 km and, after the release of radioactive materials was confirmed, some protective measures were to be implemented based on the ambient dose rates measured over the affected area. The radiological safety of those living in the affected area was judged by comparing the predicted doses with the operational intervention levels (OILs) that were administered following international standards (10). The concepts of these planning zones are illustrated in Figure 1.

The current criteria for evacuation indicated in NERG are essentially based on non-human aspects, such as the distance from the accident site, geophysical conditions, and the situation at the accident facility. This approach roots from the current recommendation made by the International Commission on Radiological Protection (ICRP) (11) such as “to manage and control exposures to ionizing radiation so that deterministic effects are prevented and the risks of stochastic effects are reduced to the extent reasonably achievable” and also to “avert discrimination.” However, according to the experiences of the Fukushima Daiichi accident, it is noteworthy that additional consideration on an individual basis is required for vulnerable people because they could easily suffer serious health consequences owing to sudden changes in their surroundings during or after their evacuation.

## 3. The process of justification

The ICRP presented three fundamental principles of radiological protection against exposure to radiation sources: justification, optimization, and application of dose limits (11). The principle of justification denotes “any decision that alters the radiation exposure situation should do more good than harm.” The optimization of protection has been defined as “the likelihood of incurring exposure, the number of people exposed, and the magnitude of their individual doses should all be kept as low as reasonably achievable, taking into account economic and societal factors.” The principle of application of dose limits denotes “the total dose to any individual from regulated sources in planned exposure situations other than medical exposure of patients should not exceed the appropriate limits specified by the Commission (ICRP).” Note that the regulatory dose limits are used by the authorities in planned exposure situations and do not apply to the emergency situations discussed in this study.

The process of justification, that is, assuring that “good” is more than “harm,” would not be easy in a large-scale nuclear accident. This process can become chaotic because many people with various views and preferences are involved. Those involved in the decision-making of protective measures that are to be taken in a nuclear emergency should have in advance a clear concept regarding the evacuation of vulnerable people so that the most appropriate decision with the maximum benefit is taken. Such preparedness from a communication aspect would ensure that the most reasonable actions are promptly implemented in emergencies.

Regardless of whether the cause is natural or factitious, death or severe disease is widely perceived as an index of harm. Based on this perception, the justification for the evacuation of vulnerable people in the Fukushima Daiichi accident is considered inappropriate for balancing the averted health risk (good) and enhanced health damage (harm). The United Nations Scientific Committee on the Effects of Atomic Radiation (UNSCEAR) estimated that an effective dose of a maximum of approximately 50 mSv was averted by the evacuation measures adopted for all residents in the 20 km zone (12). Using the nominal risk coefficient for cancers, including leukemia (0.055 per Sv for the entire population) provided by the ICRP (11), this averted dose corresponds to an averted nominal risk of 0.00275 (1 in 364). Such a small increase in radiation-induced cancers is indiscernible from cancers induced by other causes; currently, more than 20% of the Japanese population die of cancer, and the regional cancer mortality rates on a prefectural basis vary by 10% or more (13). Therefore, the physical burden experienced by more than 50 patients who died during or shortly after the evacuation was far greater than the possible increase in cancer mortality estimated if the evacuation was not implemented. Thus, the evacuation of hospitalized patients requiring continuous medical care was not justified.

**Figure 2:**
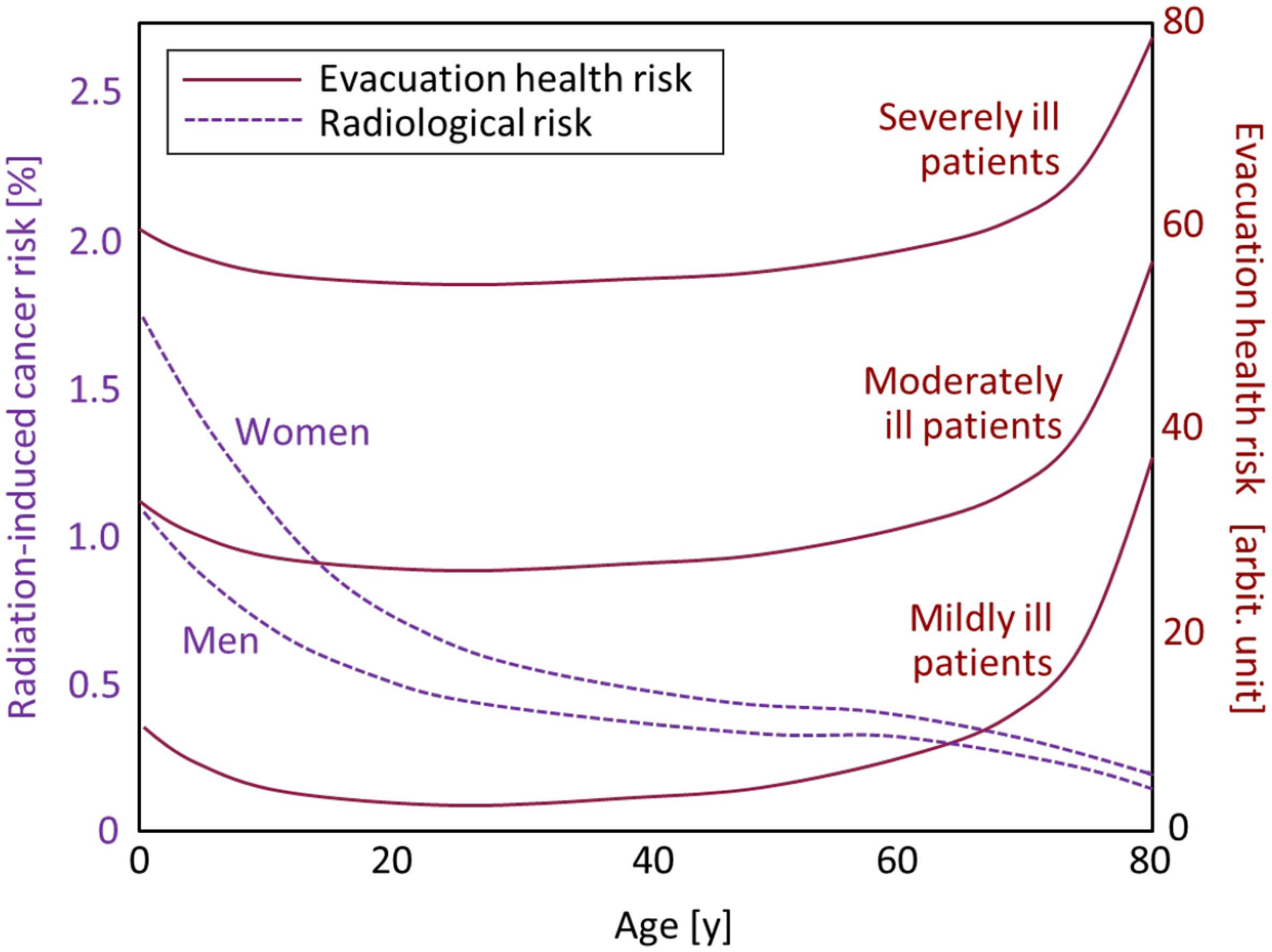
Age-dependent radiation-induced cancer risks for women and men (dotted lines) and evacuation-induced health risks of hospitalized patients (solid lines) categorized into three groups: mildly, moderately, and severely ill patients. Note that the scale of the evacuation health risk was provided for illustrative purposes, as it should be flexibly determined on a case-by-case basis according to the surrounding situation.

In the present study, the health impact of evacuation is quantitatively discussed by comparing the radiological risk, which can be expressed as the age-specific lifetime attributable risk (LAR) of radiation-induced cancer mortality provided by the BEIR VII Committee (14). Figure 2 presents the plots of LAR expressed as the increased estimated rate of cancer mortality for 100,000 men or women who received a single exposure of 0.1 Gy γ-rays at a specific age. LAR was higher in women than men over the entire age range. For both sexes, infants (age: 0 years) had the highest LAR, which rapidly decreased with growth. The radiological risk of 0.1 Gy exposure in older adults (> 65 years old) was less than 0.4%, while that for infants was more than 1.0%. The nominal risk mentioned above does not consider such differences in radiosensitivity depending on age and sex.

In contrast, the health risks caused by evacuation are considered to increase with age, as older individuals are generally more vulnerable than younger individuals. The evacuation-induced health risks of nursing home residents were estimated to be up to 30 times higher than their radiological risks (15). The evacuation-induced health risk is remarkably enhanced in hospitalized patients compared with healthy individuals, as observed in the Fukushima Daiichi accident (2, 3). In Figure 2, the age-dependent curves of evacuation-induced health risk are presented for three categories of physical conditions (mildly, moderately, and severely ill), together with the radiological risks. Note that the scale of the evacuation health risk was provided for illustrative purposes (15), as it should be flexibly determined by medical personnel on a case-by-case basis. While the curves of evacuation health risk could vary according to the health status of patients and surrounding situations, it can be pointed out that special attention is required in the evacuation of hospitalized patients who could receive significant physical damage by the discontinuation or deterioration of medical care.

## 4. Discussion

As mentioned in Section 2, the current evacuation criteria including the designation of the UPZ and PAZ are based on non-human aspects, such as the distance from the accident site and damage to the accident facility. Although this approach is convenient for treating people equally, it may be inappropriate from a viewpoint of justification. According to the experience from the Fukushima Daiichi accident, human aspects such as age and health status need to be considered to ensure that the net benefit is positive for vulnerable people who would be most significantly affected by the accident. Thus, the author presents a conceptual basis for decisions regarding the evacuation of individuals with serious health issues, typically, hospitalized patients.

The radiological safety of the people in the affected area is assessed by the regulatory authority based on the doses predicted from up-to-date information on the progress of the accident facility, radiation monitoring data, weather conditions, and geophysical features under the worst-case scenario of emissions, dispersions, and depositions of radionuclides (8). The predicted doses indicate the potential radiological risks to general population groups. This process is urgently required but difficult to achieve because reliable information for analyses can be limited in the transient, chaotic situation immediately after the occurrence of a nuclear event, and consequently the doses and risks estimated for those in affected areas can be accompanied by significant uncertainty, as confirmed by the United Nations in the assessment of the Fukushima Daiichi accident (12, 16). As the available time from the onset of a nuclear accident to the performance of protective actions would be highly limited, it is desirable to establish a guide in advance to determine the evacuation zones within a limited time and acceptable uncertainty and then to smoothly evacuate the people having a variety of lifestyles in the designated zones.

**Figure 3:**
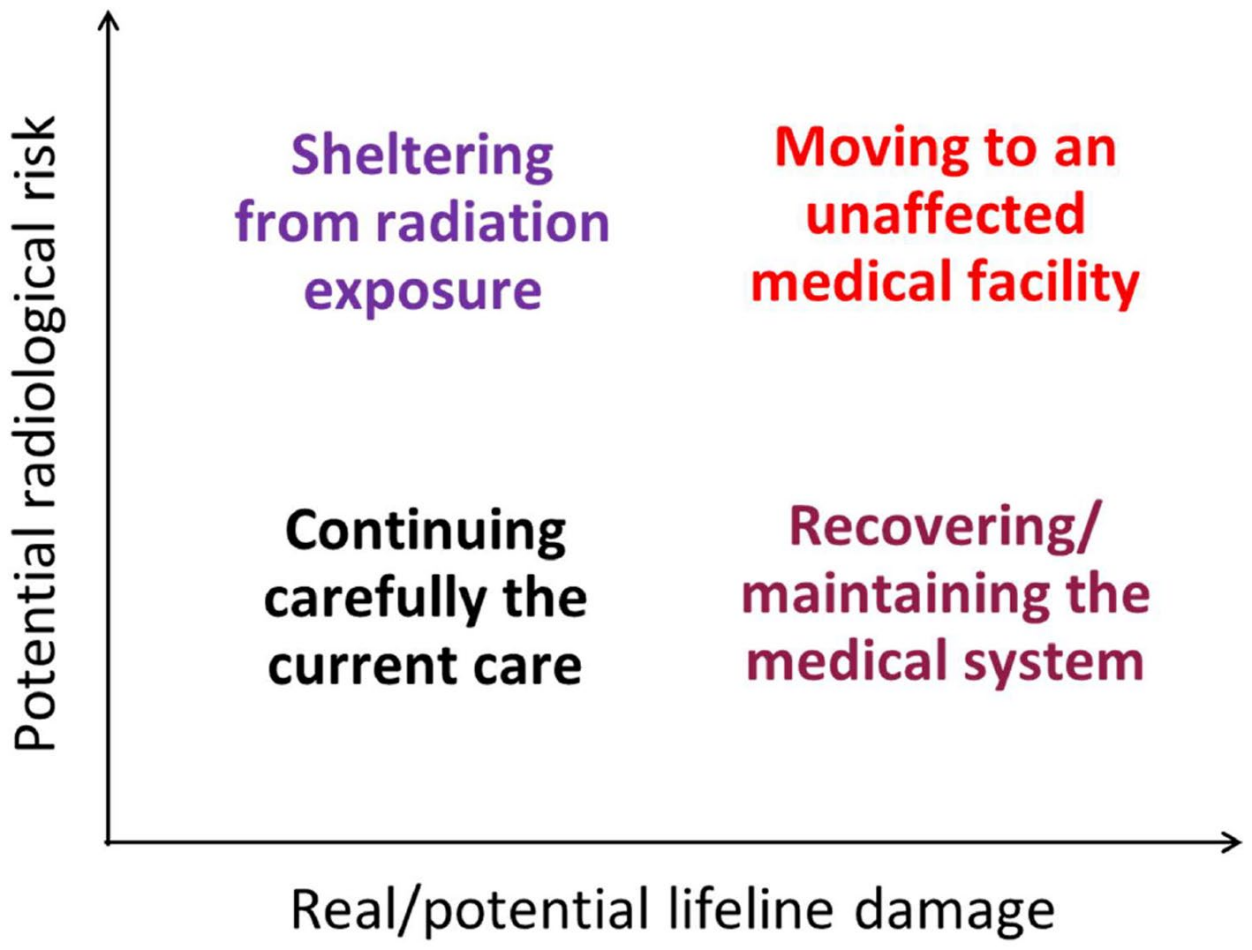
Major actions to be focused on for sustaining the medical care of hospitalized patients during nuclear emergencies under different levels of real/potential lifeline damage and potential radiological risk.

In the implementation of evacuation measures, careful attention should be paid to hospitalized patients on an individual basis to prevent deterioration of their health status. With the insight that evacuation itself could endanger the lives of such vulnerable people, the author presents a conceptual basis for decisions regarding the treatment of hospitalized patients in Figure 3. This diagram shows desirable actions for sustaining the medical care of hospitalized patients under different situations envisaged in a nuclear emergency, that is, different levels of real or potential lifeline damage (difficulty in continuing critical medical care) and potential radiological risk (predicted radiation-induced health consequences).

In cases where both lifeline damage and radiological risk are low, it will be justified to continue the current care at the same place, paying careful attention to the progressive emergent situation, since ceasing the care due to evacuation would bring about a much higher health risk than averting radiological risk.

In cases where lifeline damage is low but radiological risk is high, it is desirable to implement protective measures such as sheltering to prevent excessive radiation exposure and continue care at the same place. In a large-scale nuclear accident, sheltering generally means staying indoors to prevent the inhalation of radioactive gasses (e.g., iodine-131). Since hospitalized patients are usually indoors, additional sheltering measures are primarily meant to improve the airtightness of buildings, such as sealing windows and limiting the number of door openings. While this measure may raise concerns about possible high-dose exposure of healthcare workers (doctors, nursing staff, and caregivers), it is considered that some enhancement of the potential radiological risk would be socially accepted in urgent, life-saving activities. Concerning occupational exposure during emergencies, the ICRP has stated that no dose restrictions (i.e., no specific reference levels) should be taken in any life-saving activities if the benefit to others outweighs the risk to rescuers and informed volunteers (11), whereas the reference level ranges from 500 to 1,000 mSv for other urgent rescue operations (Table 1). The author understands that some workers may refuse to stay in the evacuation zone because of a strong fear of radiation exposure, and in such situations, decisions should be flexibly made with respect to individual differences in risk perception.

**Table 1:** The reference dose levels recommended by ICRP for protection criteria on occupational exposure in emergency exposure situations (11).

Categories of exposure	Reference levels (in effective dose)
Life-saving (informed volunteers)	No dose restrictions if the benefit to others outweighs the risk to rescuers
Other urgent rescue operations	1,000 or 500 mSv
Other rescue operations	< 100 mSv

In cases where lifeline damage is high but radiological risk is low, it would be worthwhile to try to prioritize maintaining or recovering the medical system for continuing the current care at the same place. In this case, the recovery of damaged medical facilities, including the supply of various resources, such as medical devices and healthcare workers, must be assisted both technically and financially under the supervision of the local government. During this lifeline recovery process, hospitalized patients should be treated with caution.

In cases where both the levels of lifeline damage and radiological risk are too high to maintain current care, it would be justified to move all hospitalized patients, even those who need continuous and intensive medical care, to medical facilities outside the evacuation zone. In this evacuation process, careful attention should be paid to the conditions of moving patients because their lives can easily be threatened by changes in medical circumstances. Particularly when the critical infrastructure for medical services is disrupted, evacuation of hospitalized patients with necessary medical resources will be complicated and challenging (17, 18). The ability to provide adequate medical care for all patients throughout disruption needs to be evaluated as immediately as possible and, in case the infrastructure damage is found to be so severe that adequate care cannot be provided for the entire population of patients, the medical personnel needs to decide which patients should be prioritized for evacuation and what resources are required to ensure the adequate care for those being evacuated.

In any of the cases above, it will always be necessary to minimize the physical and mental stress of hospitalized patients by carefully monitoring the situations surrounding the patients, considering that the situation could notably change in a short time. It should be noted that, soon after a large-scale natural disaster, the health status of vulnerable people can easily be exacerbated owing to deteriorated living conditions such as malnutrition, unsanitary environment, and shortage of necessary resources. If the primary resources such as medical staff, utilities, and medication for hospitalized patients are insufficient, evacuation should be urgently decided so that critical resources for patients would be promptly allocated most appropriately. Collaborative participation of external bodies such as local and national governments, rescue services, emergency medical facilities, relevant research organizations, non-governmental groups, and other stakeholders would play a key role in achieving the most successful evacuation of vulnerable people while maintaining their clinical statuses (19–21). In addition, possible crowding during a displacement would increase the risk of infectious disease transmission, as observed in the recent major earthquakes in Japan (22, 23). When the status of a damaged nuclear facility worsens, followed by possible additional radioactive releases to the living environment, such unstable conditions in nuclear emergencies can cause significant stress to patients and notably exacerbate their health statuses. Considering these, the decision-making on the measures for hospitalized patients (Figure 3) should be carefully made but done so immediately to maximize a net benefit, that is, to minimize the number of victims overall.

This brief research report intends to provide a conceptual basis for the evacuation of hospitalized patients during a nuclear emergency, based on the lessons learned from the Fukushima Daiichi accident. The author understands that it is not simple to realize this concept in a real situation, where judgments can be split among healthcare workers according to their diverging perceptions of different types of risks. Nevertheless, the author expects that this brief report will provide some helpful insights to the stakeholders involved in the preparedness and responsiveness for nuclear emergencies, fostering discussions among them about how to take the most appropriate decisions and actions on the evacuation of all inpatients considering the principle of justification. An example of the topics to be discussed is the constant preliminary assessments of medical special need (MSN) levels of individual patients that would be effective, even for quick assessment in a nuclear emergency (18). Further investigations including the development of a more comprehensive guidance should be performed to achieve practical implementations of the presented conceptual basis while becoming acquainted with diverging perceptions and changing opinions among various stakeholders.

## Data availability statement

The original contributions presented in the study are included in the article/supplementary material, further inquiries can be directed to the corresponding author.

## Author contributions

HY conceptualized the study, performed the analysis, and prepared and revised the article.

## Funding

This study was supported in part by the Program of the Network-type Joint Usage/Research Center for Radiation Disaster Medical Science funded by the Ministry of Education, Culture, Sports, Science, and Technology of the Japanese Government.

## Acknowledgments

Sincere appreciation is expressed to Sergey Shinkarev (State Research Center, Burnasyan Federal Medical Biophysical Center of Federal Medical Biological Agency, Russia) and Dean Kyne (University of Texas, Rio Grande Valley, USA) for their helpful advice during discussions on evacuation issues. I would like to thank Editage for English language editing.

## Conflict of interest

The author declares that the research was conducted in the absence of any commercial or financial relationships that could be construed as a potential conflict of interest.

## Publisher’s note

All claims expressed in this article are solely those of the authors and do not necessarily represent those of their affiliated organizations, or those of the publisher, the editors and the reviewers. Any product that may be evaluated in this article, or claim that may be made by its manufacturer, is not guaranteed or endorsed by the publisher.
